# Solar Photocatalytic Activity of Ba-Doped ZnO Nanoparticles: The Role of Surface Hydrophilicity

**DOI:** 10.3390/nano13202742

**Published:** 2023-10-10

**Authors:** Abdessalem Hamrouni, Marwa Moussa, Nidhal Fessi, Leonardo Palmisano, Riccardo Ceccato, Ali Rayes, Francesco Parrino

**Affiliations:** 1 Laboratory of Catalysis and Materials for the Environment and Processes LRCMEP (LR19ES08), Faculty of Sciences of Gabès, University of Gabès, University Campus Erriadh City, Gabès 6072, Tunisia; hamrouni-28@hotmail.fr (A.H.); moussaamarwaa@gmail.com (M.M.); nidhal_fessi@hotmail.fr (N.F.); ali.rayes@fsb.rnu.tn (A.R.); 2 Department of Chemical Engineering-Processes, National Engineering School of Gabès, Omar El Khateb Avenue, Zrig, Gabes 6029, Tunisia; 3 Laboratoire d’Automatique et de Génie des Procédés (LAGEPP), University of Lyon, UMR 5007 CNRS, University Claude Bernard Lyon 1, 69622 Villeurbanne, France; 4 Department of Engineering, University of Palermo, Viale Delle Scienze Ed. 6, 90128 Palermo, Italy; leonardo.palmisano@unipa.it; 5 Department of Industrial Engineering, University of Trento, via Sommarive 9, 38123 Trento, Italy; riccardo.ceccato@unitn.it

**Keywords:** Ba-doped ZnO, hydrophilicity, solar photodegradation, 4-nitrophenol, photocorrosion suppression

## Abstract

Bare zinc oxide (ZnO) and Ba-doped ZnO (BZO) samples were prepared by using a simple precipitation method. The effects of Barium doping on the structural, morphological, and optoelectronic properties, as well as on the physico-chemical features of the surface were investigated and correlated with the observed photocatalytic activity under natural solar irradiation. The incorporation of Ba^2+^ ions into the ZnO structure increased the surface area by ca. 14 times and enhanced the hydrophilicity with respect to the bare sample, as demonstrated by infrared spectroscopy and contact angle measurements. The surface hydrophilicity was correlated with the enhanced defectivity of the doped sample, as indicated by X-ray diffraction, Raman, and fluorescence spectroscopies. The resulting higher affinity with water was, for the first time, invoked as an important factor justifying the superior photocatalytic performance of BZO compared to the undoped one, in addition to the slightly higher separation of the photoproduced pairs, an effect that has already been reported in literature. In particular, observed kinetic constants values of 8∙10^−3^ and 11.3∙10^−3^ min^−1^ were determined for the ZnO and BZO samples, respectively, by assuming first order kinetics. Importantly, Ba doping suppressed photocorrosion and increased the stability of the BZO sample under irradiation, making it a promising photocatalyst for the abatement of toxic species.

## 1. Introduction

Human societies are finally aware of the need to use new technologies to reduce the climate crisis and avoid environmental disasters that have been occurring quite often in recent years. Therefore, the problem of environmental remediation using efficient and environmentally friendly techniques is a hot topic today [1]. The photocatalytic process conducted under solar irradiation is a promising candidate that satisfies the need for efficiency and safety. Its economic convenience is mainly based on the possibility of using nanomaterials not only with high performance, but also with good (photo)stability under the operating conditions [2,3]. Currently, only about 0.014% of solar energy (3.85∙10^24^ J∙year^−1^) impinging on the Earth, and which is absolutely free, is exploited to meet the demand for renewable energy sources and for the solution of environmental problems [4]. Therefore, the conversion of sunlight has become one of the major goals of scientists. The activation of semiconducting materials by sunlight results in the formation of electron-hole pairs, which in turn produce an electric current or trigger useful photocatalyzed chemical reactions on their surface. In this way it is also possible to carry out up-hill reactions that simulate those occurring in nature, which store solar energy in simple chemical bonds [5,6]. It is also possible to remove dangerous compounds through their total photo-mineralization or to synthesize organic substances with high added value in a green and safe way, starting from compounds of little economic interest [7,8].

Zinc oxide (ZnO) and titanium dioxide (TiO_2_) in the form of nanomaterials are maybe the most investigated and applied photocatalysts traditionally proposed for wastewater remediation, due to their low cost and good optical, electronic, and structural properties [9,10,11,12,13,14]. However, both semiconductors scarcely absorb sunlight and have a high rate of electron/hole recombination (e^−^/h^+^). ZnO, exclusively, is far less stable than TiO_2_ due to photocorrosion phenomena [15,16,17]. To overcome these drawbacks, it has been variously modified. Usually, sensitization to sunlight and greater stability have been obtained by doping ZnO with cationic or anionic elements, combining with other semiconductors or carbon-based materials, or decorating with noble metal particles [18,19,20]. Specifically, introducing alkaline earth metals into ZnO has emerged as a promising approach to develop new doped photocatalysts with distinctive optoelectronic properties [21,22]. Numerous studies have highlighted the potential of Mg, Ba, Ca, and Sr doping in enhancing ZnO photoactivity under both artificial UV light and visible light irradiation [23,24,25,26,27]. However, investigations under natural sunlight irradiation have been very limited. To the best of our knowledge, no studies have specifically addressed the effects of the doping on the surface features of the doped ZnO photocatalysts, and specifically on the induced hydrophilicity. Understanding the water molecules’ affinity to the photocatalyst’s surface is vital for photodegradation reactions, as it significantly influences the generation of the reactive species such as hydroxyl radicals (OH^●^) [28]. 

Prior research efforts on alkaline earth metal-doped ZnO, especially with barium, have primarily focused on investigating optoelectronic properties, surface area, and morphology. In recent studies, Bhamare et al. discovered that Ba-doped ZnO samples exhibit remarkable efficiency in the photocatalytic mineralization of Linezolid antibiotics when irradiated under UV light. Various experimental parameters such as pH medium, light intensity, and photocatalyst quantity were explored, and the superior photocatalytic performance was primarily attributed to the enhanced separation of e^−^/h^+^ pairs [29]. Similarly, in another study, Jayakrishnan et al. demonstrated the photocatalytic activity of Ba-doped ZnO in the photodegradation of Rhodamine B under visible light. This performance was attributed to the narrow band gap and the presence of oxygen vacancies [30]. Behnaz et al. verified that the photocatalytic efficacy of Ba-doped ZnO in the photodegradation of Rhodamine B is mainly due to the inhibition of the recombination rate under UV light, while, under visible light irradiation, indirect photocatalytic mechanisms involving the excited adsorbed dye molecules had a pivotal role [31]. Qiu et al. [23] stated that the replacement of Zn^2+^ ions with Mg^2+^ contributes to e^−^/h^+^ separation, resulting in increased photodegradation of methylene blue under UV light irradiation. Opposite results were found, however, when Mg^2+^ ions were inserted into interstitial sites with a doping percentage higher than 4%, because the higher conduction band (CB) potential reduced the light harvesting ability [23]. Elhalil et al. [24] reported that the photodegradation of caffeine occurred more rapidly using the 5% Ca–ZnO–Al_2_O_3_ system than bare ZnO and ZnO–Al_2_O_3_ systems. To explain this result, the better crystallinity of the photocatalyst was invoked, as well as its greater ability to absorb UV light with a consequent higher formation of e^−^/h^+^ pairs. Modvi et al. [25] found that the insertion of Ba^2+^ ions into the ZnO lattice shifted the absorption into the wavelength range of visible light, reducing the phenomenon of e^−^/h^+^ recombination and improving the photocatalytic efficiency. The introduction of Ca^2+^ ions can also reduce the band gap energy (E_g_), as described by Irshad et al. [26]. Similarly, Oliveira et al. found that the photoactivity of ZnO can be boosted by doping with Ca^2+^, due to the crystal lattice changes caused by its presence and the role of this species as electron trap [27].

The above results demonstrate that the alkaline earth metals are promising as far as the improvement of the optoelectronic features of ZnO-based photocatalysts are concerned. However, to the best of our knowledge, the impact of this modification on the surface properties, and in particular on hydrophilicity, has not been adequately considered. In fact, the photocatalytic reactions take place on the semiconductor surface and the peculiar interaction between the surface and the substrate is a key parameter to be considered. In this investigation, some structural, morphological, optical, electronic, and surface features of Ba-doped ZnO are reported, and particular attention is devoted to the influence of the presence of barium on the surface properties of ZnO. The characterization results were correlated with the higher photocatalytic activity under solar irradiation and the higher stability of the doped material compared with the bare one against photocorrosion of ZnO, which often discourages the possible applications of this oxide especially in aqueous systems.

## 2. Results and Discussion

**Figure 1:**
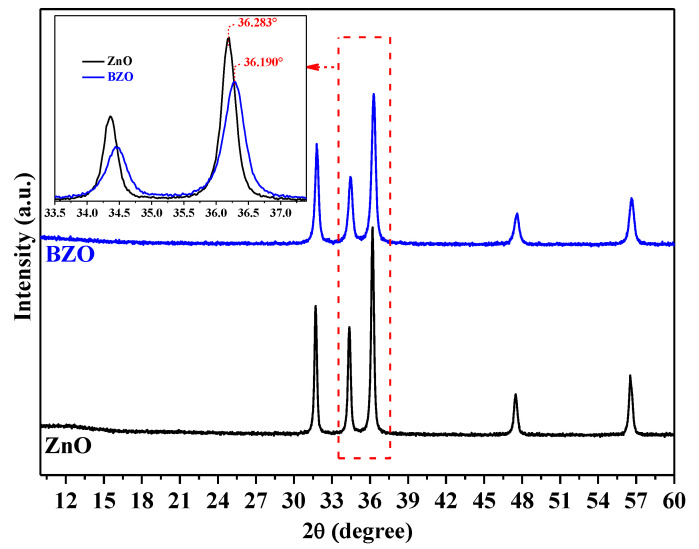
XRD patterns of the undoped ZnO (black) and BZO (blue) photocatalysts and (inset) the (002) and (101) peaks’ shift.

The XRD patterns of the synthesized bare ZnO and BZO photocatalysts are presented in Figure 1. The diffraction peaks of the ZnO and BZO samples conform to the hexagonal phase of zincite, as verified by JCPDS data (Card No. 36-1451). No other barium oxide peaks or barium-containing species were detected, confirming the incorporation of Ba^2+^ ions into the ZnO lattice. The radius of Ba^2+^ (1.35 Å) prevents the substitution of the Zn^2+^ ion, which has a smaller radius (0.74 Å). Consequently, as previously suggested [32], Ba^2+^ ions can occupy interstitial sites within the ZnO lattice. This hypothesis was verified by determining the lattice parameters. Indeed, the inset of Figure 1 clearly reveals that the presence of Ba^2+^ dopant led to a relative shift in the 2θ values of +0.093°. As a result, the parameters (a) and (c) of ZnO increased from 3.2501 and 5.2068 to 3.2519 and 5.2098 Å, respectively. Therefore, the volume (V) of the unit cell increased from 47.633 to 47.714 Å^3^, indicating the inclusion of Ba^2+^ ions (see Table 1).

Table 1 displays the values of the mean crystallite size (D), along with the specific surface area (SSA) and the band gap energy values (E_g_) of the samples. After the introduction of Ba dopant, the mean crystallite size (D) of ZnO decreased from 36.4 to 23.0 nm. This reduction in crystallite size due to Ba doping aligns with recent research [30]. The error values for (a) and (c) are indicated by placing the last digit in brackets.

**Figure 2:**
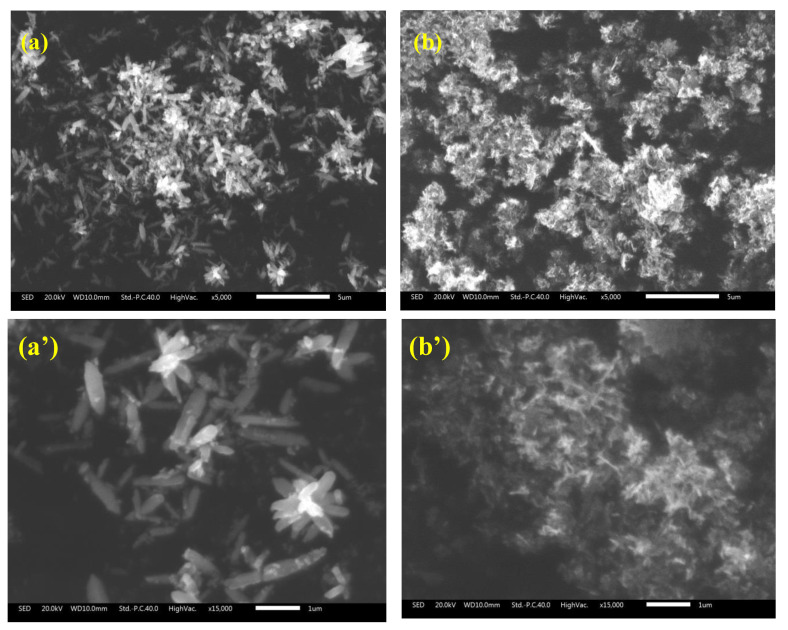
SEM images of bare ZnO (**a**,**a’**) and BZO (**b**,**b’**) photocatalysts at different magnifications.

Figure 2 shows the SEM micrographs of the photocatalysts. It can be seen that both bare ZnO and BZO nanoparticles possess a nanowire shape and are quite homogeneous in size. However, the doped BZO particles are much smaller. The incorporation of Ba^2+^ ions into the ZnO lattice prevents grain growth, in agreement with XRD results and related literature [24,33]. 

**Figure 3:**
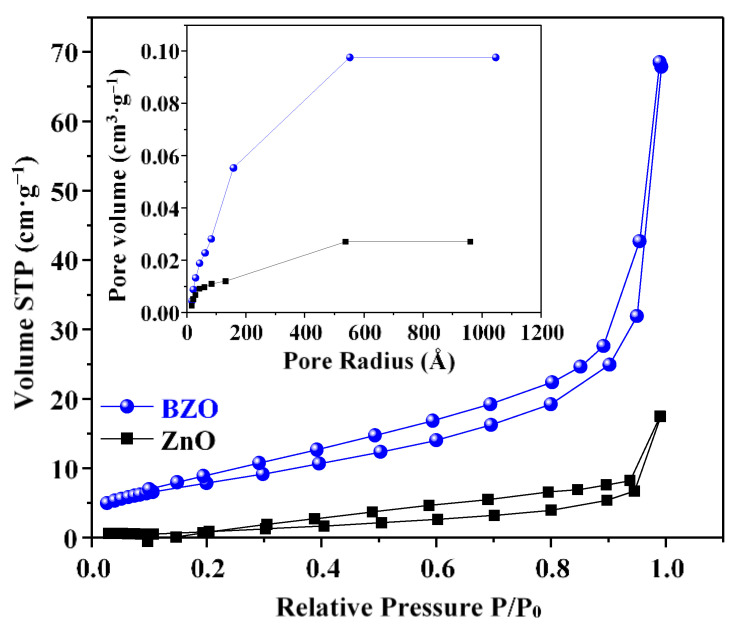
Nitrogen adsorption–desorption isotherms and (inset) pore size distribution of bare ZnO (black squares) and BZO (blue circles) photocatalysts.

The adsorption and desorption isotherms and the pore size distribution of the bare ZnO and BZO samples are presented in Figure 3. The isotherms of the synthesized samples are type II, as generally observed in non-porous solids. The H3 type hysteresis is typical of aggregates with formation of interparticle voids [33]. This is in accordance with the small nanoparticle size retrieved by XRD and SEM analysis. 

**Figure 4:**
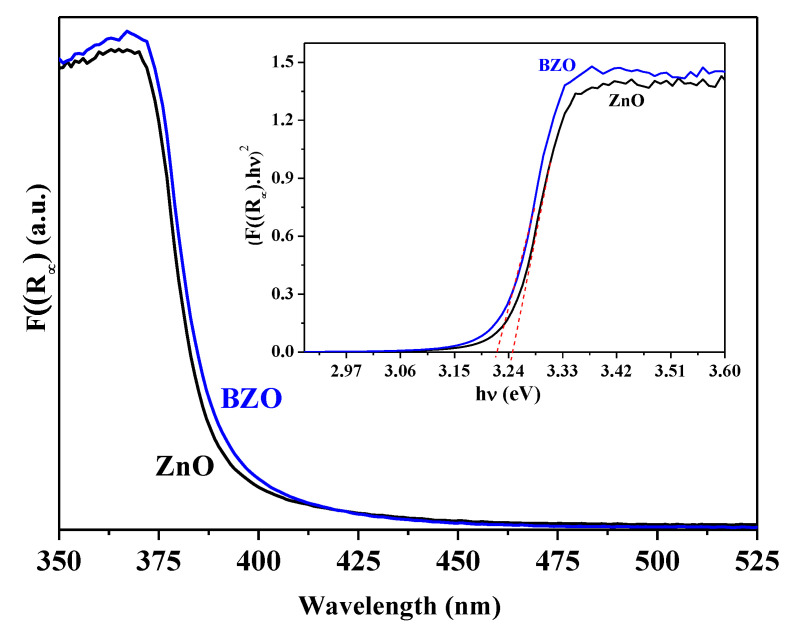
UV-vis spectra and (inset) Tauc plots of ZnO (black) and BZO (blue) photocatalysts.

Diffuse reflectance spectra (DRS) of bare ZnO and BZO samples were recorded in order to explore how Ba doping could affect the optoelectronic properties of the ZnO photocatalyst (Figure 4). The spectrum of BZO is slightly redshifted with respect to bare ZnO. The inset reports Tauc plots showing E_g_ values that are equal to 3.25 and 3.22 eV for bare ZnO and BZO samples, respectively.

It is evident that the insertion of Ba^2+^ ions only slightly affects the optical properties of ZnO. The slight decrease in the E_g_ value has been explained in the relevant literature by the inclusion of intermediate energy levels below the CB of ZnO which led to better e^−^/h^+^ separation. These levels can be related to the influence of the 4d orbitals of Ba atoms that narrow the E_g_ [30]. However, the slight narrowing of E_g_ observed upon Ba doping in accordance with the literature [25,30,31] is unlikely to account for the relevant changes in the photocatalytic activity (see below). For this reason, we further investigated the surface properties of the samples, considering that the surface is where the photocatalytic reactions take place. 

**Figure 5:**
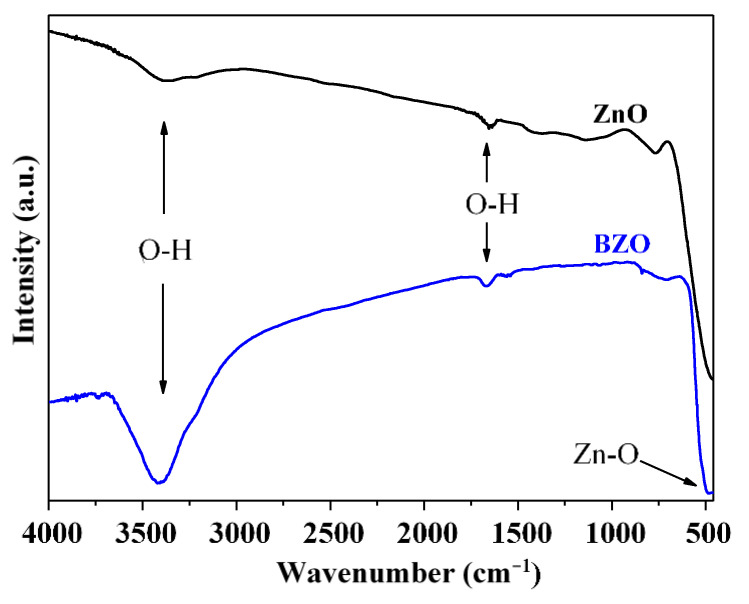
Normalized FTIR spectra of ZnO and BZO photocatalysts.

To study surface functional groups, FTIR spectra of ZnO and BZO samples were collected in the wavenumber range 450–4000 cm^−1^. The spectra were normalized by considering the vibration of the Zn–O bond at about 500 cm^−1^, to obtain semi-quantitative information. As illustrated in Figure 5, the broad bands detected at 3340–3650 and 1640 cm^−1^ can be attributed to vibrations of the O–H bond of adsorbed water molecules or surface hydroxyl groups [34]. Notably, the bands are clearly smaller in the case of the ZnO sample [35,36,37]. This observation suggests that the incorporation of Ba into the ZnO lattice increases the affinity of the surface for water molecules, thus endowing it with a pronounced hydrophilic character.

**Figure 6:**
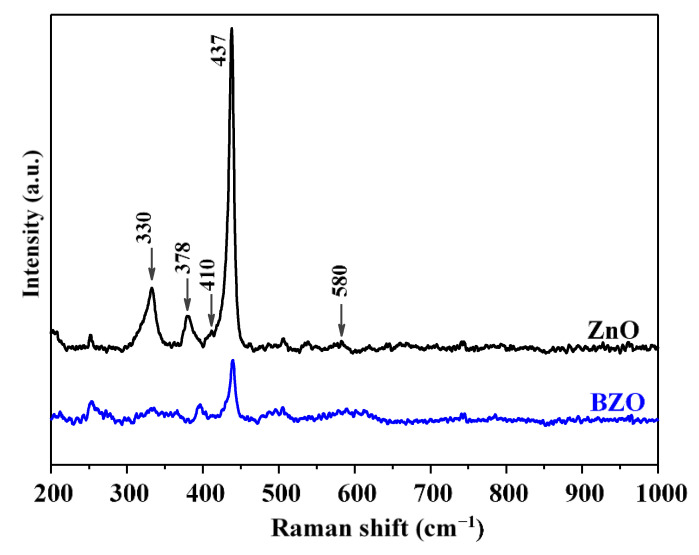
Raman spectra of bare (ZnO, black line) and Ba-doped (BZO, blue line) ZnO photocatalysts.

The Raman spectra of bare and Ba-doped ZnO samples are provided in Figure 6. The band at 437 cm^−1^ is characteristic of the hexagonal zincite structure of ZnO and it is due to the optical phonon E_2_ (high) [38]. The A_1_ longitudinal optical (LO) mode appears at 331 cm^−1^, the vibrational activity of A_1_ transverse optical (TO) mode at 378 cm^−1^, and the E_1_ (TO) at 410 cm^−1^. These findings are in good accordance with relevant literature [37,38]. 

BZO sample shows a broad signal at 580 cm^−1^ which is slightly visible in the spectrum of bare ZnO. This band is attributed to multiphonon scattering processes and has been correlated in the literature with defectivity such as oxygen vacancies [28].

The intensity of the peaks of ZnO is higher with respect to the BZO sample. However, the relative intensity of the peak at 330 cm^−1^ with respect to the main band at 437 cm^−1^ is higher, even if broader, in the BZO sample. Moreover, as previously observed, the peak at 580 cm^−1^ appears clearly in the doped sample and is negligible in ZnO. The higher intensity of the latter bands, associated with longitudinal optical (LO) modes, indicates that the presence of Ba^2+^ into the lattice of ZnO introduces novel defectivity. Oxygen vacancies have often been identified as responsible for enhanced water adsorption [28,39]. Moreover, the adsorption of hydroxyl groups into Zn^2+^ sites has been demonstrated to be kinetically favored with respect to oxygen adsorption [40]. Therefore, the defectivity induced by the presence of barium can be related with the higher hydrophilicity observed by FTIR spectroscopy.

**Figure 7:**
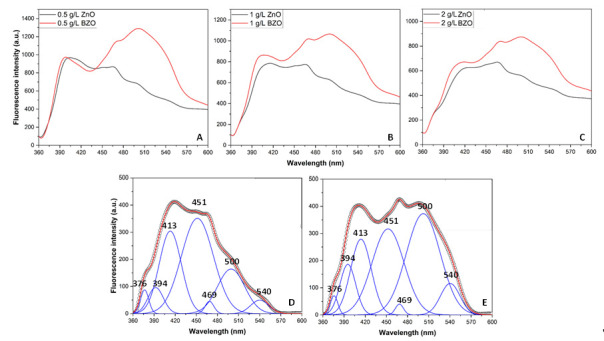
Normalized emission spectra (λ_exc_ = 325 nm) of 0.5 (**A**), 1.0 (**B**), and 2.0 g∙L^−1^ (**C**) water suspensions of bare ZnO (black lines) and BZO (red lines). (**D**,**E**) report the deconvoluted ZnO and BZO spectra, respectively, for 2.0 g∙L^−1^ suspensions, obtained upon baseline correction.

The higher defectivity of the BZO sample can be further highlighted by comparing its fluorescence spectrum with the one of bare ZnO. Figure 7 shows the spectra acquired on different ZnO and BZO suspensions, i.e., 0.5, 1.0, and 2.0 g∙L^−1^, reported in Panels A, B, and C, respectively. Panels D and E report the deconvoluted components of the ZnO and BZO spectra, respectively, obtained for 2.0 g∙L^−1^ suspensions.

The emissive behavior of ZnO and BZO samples is similar in the range between 360 and 430 nm, and the recorded intensity in this region only slightly changes upon increasing the amount of dispersed photocatalyst (A–C). Deconvolution in this region highlights three main contributions at 376, 394, and 413 nm. The first two bands can be attributed to band-to-band exciton radiative recombination and band edge emissions [41], while the violet emission at 413 nm can be ascribed to electron transitions from shallow donor levels of neutral interstitial Zn defects (Zn_i_) close to the conduction band [42]. The luminescence spectra of ZnO and BZO become significantly different between 430 and 600 nm, where the emission of the BZO sample is stronger than the one of ZnO. Notably, the intensity of the bands in this region decreases with increasing amounts of sample dispersed in water (A–C), possibly due to scattering, trivial energy transfer, or quenching phenomena. Deconvolution in this region highlights four bands at 451, 469, 500, and 540 nm. The blue emission at 451 nm has been attributed to electron transitions to shallow levels of Zn vacancies defects (Zn_V_) close to the valence band. The redshifted emission at 469 nm can be related to electron transitions from interstitial Zn (Zn_i_) to Zn vacancy (Zn_V_) defects levels [42]. Finally, the two green emissions at 500 and 540 are typical fluorescence bands related to the presence of oxygen vacancies [43] and interstitial oxygen sites [41], respectively. Notably, these two components show higher relative intensity in the BZO sample with respect to the ZnO one (D–E), thus confirming the higher defectivity of BZO induced by the presence of barium, in agreement with XRD and Raman results.

**Figure 8:**
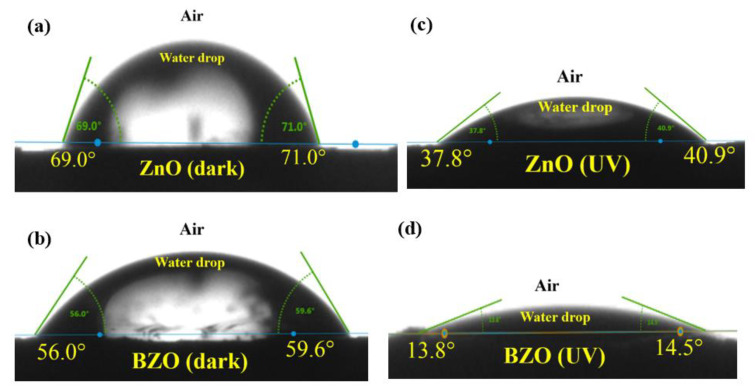
Shape of water drop on ZnO and BZO pellets for static contact angle measurements in the dark (**a**,**b**) and immediately after 10 min exposure to UV light irradiation (**c**,**d**).

To further confirm the increased hydrophilicity of the BZO sample, contact angle measurements were performed. Figure 8 shows the static contact angles measured between the deposited water droplet and the surface of the ZnO and BZO photocatalysts in the dark (a and b) and after 10 min of exposure to UV light irradiation (c and d).

Regarding the BZO photocatalyst, it is evident that the water droplet exhibits a flatter shape in both dark and UV light conditions when compared to the bare ZnO sample. The images clearly demonstrate that the contact angles of ZnO are notably higher than those of BZO photocatalyst under identical conditions, particularly when exposed to UV light. Consequently, BZO sample exhibits a more pronounced hydrophilic character, which is consistent with FTIR and Raman analyses.

Similar results were reported for ZnO nanorods which showed higher wettability due to surface roughness [44]. Mg-doped ZnO, in which, unlike barium, Mg ions were substitutionally positioned within the ZnO lattice, also exhibited an enhanced hydrophilic character [45]. The authors accounted for the higher hydrophilicity of Mg-doped ZnO under dark conditions with the more pronounced roughness of the modified samples. This explanation, along with the novel defectivity hereby observed, may also hold for the barium-modified ZnO in the dark, considering the different surface topography shown in the SEM analyses (see Figure 2).

It is important to mention that the heightened adsorption of water molecules significantly amplifies the generation of hydroxyl radicals via oxidation of water induced by holes (h^+^) at the valence band (VB), as reported below (Equation (1)) [46,47].

H_2_O + h^+^_VB_ → H^+^ + HO^•^ (1)

**Figure 9:**
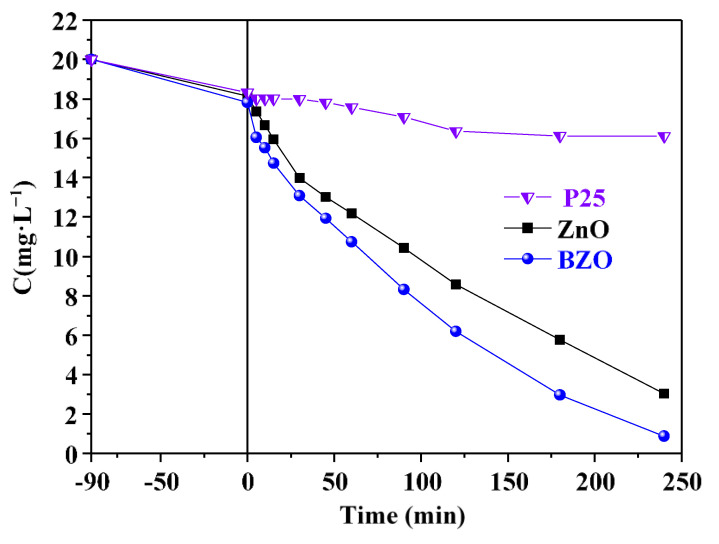
Concentration of 4-NP during photocatalytic runs under natural solar light in the presence of ZnO (■), BZO (**●**), and P25 (⧨). Irradiation started at time 0.

For this reason, there is general consensus on the direct relationship between higher hydrophilicity and photocatalytic activity [48,49]. To confirm this hypothesis, photocatalytic degradation of 4-nitrophenol (4-NP) as a model pollutant were performed under solar radiation. The results are shown in Figure 9.

Under solar light irradiation, the BZO sample demonstrated the highest efficiency compared to both bare ZnO and TiO_2_ P25. In particular, both ZnO-based samples showed superior performances compared to P25 under sunlight irradiation, and the doped sample stood out for the higher performance. Observed kinetic constant values of 8∙10^−3^ and 11.3∙10^−3^ min^−1^ were determined for the ZnO and BZO samples, respectively, by assuming first order kinetics. The higher activity of the BZO sample cannot be explained simply by considering its slightly redshifted absorption spectra and improved e^−^/h^+^ separation, as invoked in previous reports [29,30,31]. The significantly higher SSA in the BZO sample suggests a greater number of catalytically active sites. However, as presented in Figure S1, under dark conditions, the adsorbed quantity of 4-NP showed was similar for both photocatalysts. In fact, even though the BZO photocatalyst exhibited a SSA about 14 times larger than ZnO, a similar amount of 4-NP was adsorbed onto bare ZnO (ca. 11%) and BZO (ca. 15%). The adsorption results indicate that the SSA is not the primary factor influencing the BZO’s photocatalytic performance. On the other hand, it is important to note that the BZO sample exhibits a higher degree of defects, which can justify its remarkable surface hydrophilicity as observed by FTIR analyses and contact angle measurements. This aspect, often overlooked in previous studies, may have a notable impact on the photocatalytic activity. In fact, several previous studies reported the mechanism pathway of 4-NP photodegradation and showed that the degradation was mainly triggered by HO^•^ radicals (see Equation (2)), which induced poly-hydroxylation of the aromatic ring followed by its opening and, eventually, total mineralization [50,51]. 

**Figure 10:**
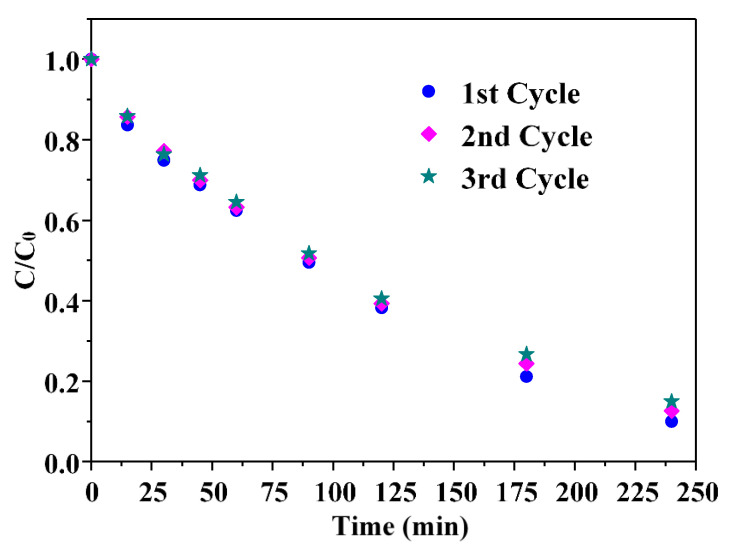
Reusability tests of the BZO photocatalyst under natural solar irradiation.

To verify the effect of Ba doping on the stability of ZnO, reusability tests were performed (Figure 10).

The progress of commercial and industrial-scale photocatalytic applications of ZnO remains hindered primarily by the adverse effects of photocorrosion, especially in aqueous systems. This fact strongly discourages the use of bare ZnO under both UV irradiation and natural solar irradiation [52]. Zhang et al. [53] observed a dramatic decrease in ZnO activity after a period of UV irradiation ranging from a few hours to a month. Yu et al. [54] showed an inactivation of ZnO of about 50% after 60 min of irradiation, while, more recently, Le et al. [55] demonstrated a ZnO weight loss of 22.3% at pH 3, 4.2% at pH 7, and 2.5% at pH 11 after five days of UV irradiation. Anodic photocorrosion of ZnO leads to the evolution of O_2_ accompanied by the dissolution of Zn^2+^ ions, according to the following reactions (Equations (2)–(4)) [16,56]:

O^2−^_surface_ + h^+^_VB_ → O^−^_surface_ (2)

O^−^_surface_ + O^2−^_surface_ + 3h^+^_VB_ → O_2(g)_ (3)

Zn^2+^_surface_ → Zn^2+^_aq_ (4)

The three tests reported in Figure 9 consistently exhibited similar performances in successive runs, indicating the remarkable stability of barium-doped ZnO. Introducing the Ba element into ZnO clearly reduces, or even suppresses, photocorrosion phenomena, aligning with findings from previous studies on the effect of alkaline earth metals dopants [52]. Furthermore, the enhanced surface hydrophilicity may also explain the stability of the BZO sample. It can be hypothesized that the oxidation of water favoured in the BZO sample may actually compete with the lattice oxidation of oxygen induced by the photogenerated holes in the bare ZnO sample. 

## 3. Materials and Methods

### 3.1. Preparation of the Photocatalysts

ZnO and BZO samples were synthesized through a straightforward precipitation method in bi-distilled water as solvent. Zinc nitrate (Zn(NO_3_)_2_·6H_2_O, 98%, Sigma-Aldrich, St. Louis, MI, USA) and barium chloride (BaCl_2_·2H2O, ≥99%, Fisher Scientific, Hampton, NH, USA) were used as the initial materials for Zn and Ba elements, respectively, without undergoing additional treatment. Ammonium hydroxide (NH_4_OH, 28%, Sigma-Aldrich) was used as the precipitating agent, with oxalic acid (C_2_H_4_O_2_, 98%, Sigma-Aldrich) as an additive. Bare ZnO was synthesized by dissolving 0.05 moles of Zn(NO_3_)_2_·6H_2_O in 500 mL of water under vigorous stirring. Then, 50 mL of C_2_H_4_O_2_ (0.1 M) were added to the starting solution at 60 °C. The pH was increased to 7 by adding a 28% NH_4_OH solution dropwise, then the precipitates were filtered and washed several times. To verify the effectiveness of the washing procedure, the conductivity of the filtrates was measured after each washing cycle using a professional benchtop meter AD3000 Conductivity-TDS-TEMP. Subsequently, the wet precipitates were placed in an electric desiccator set at 110 °C for 12 h. The Ba-doped ZnO photocatalyst, labeled herein as BZO, was prepared using the same procedure, but also adding the Ba^2+^ salt to the starting solutions. The amount of Ba and Zn precursors used was calculated so as to obtain a Zn/Ba molar ratio equal to 99/1. The final photocatalysts were obtained through a final calcination in a programmed muffle furnace for 2 h at 600 °C.

### 3.2. Characterization

A Bruker D8 Advance diffractometer was used to perform X-ray diffraction (XRD) analysis for the structural studies. The Cu Kα radiation source operated at 40 kV as voltage and the scanning range of two theta (2θ) was between 10° and 60°. The average size of the crystallites (D) was obtained using the formula of Debye Scherrer presented in Equation (5) [57]:

(5) $$D=\frac{\left(0.9\lambda \right)}{\left(\beta \cos\theta \right)}$$

where λ = 0.15405 nm is the wavelength of the X-ray, β represents the integral breadth, and θ corresponds to the Bragg’s diffraction angle. The values of the cell parameters *a* and *c* have been calculated by using the following Equation (6) for hexagonal lattice:

(6) $$\frac{1}{d^{2}\left(\mathrm{hkl}\right)}=\frac{4}{3}\frac{\left(h^{2}+k^{2}+\mathrm{hk}\right)}{a^{2}}+\frac{l^{2}}{c^{2}}$$

where *h*, *k*, and *l* are the Miller indices of spacing d_(hkl)_. 

The cell volume has been calculated according to Equation (7)

(7) $$V=\frac{\surd 3a^{2}c}{2}.$$

The diffuse reflectance spectra (DRS) were recorded from 190 to 800 nm by means of a Perkin Elmer Lambda 950 UV-vis spectrophotometer and barium sulfate (BaSO_4_, Sigma Aldrich, p.a.) was used as the reference. The conversion of the reflectance (R_∞_) to F(R_∞_) values was carried out according to the Kubelka–Munk theory. Notably, by assuming the scattering coefficient as wavelength independent, the F(R_∞_) is proportional to absorbance. The samples were assumed to be direct semiconductors. The values of the band gap energy (E_g_) were determined from the plots of [F(R_∞_)∙hν]^2^ vs. the exciting light energy (hν). E_g_ values (eV) of the ZnO and BZO samples were determined using *x*-axis extrapolation of the linear parts of the plots.

Scanning electron microscopy (SEM) observations were performed using a Jeol 6700 F FE-SEM system. A Bruker Alpha II FTIR spectrophotometer was used to record Fourier transform infrared (FTIR) spectra. A Micromeritics ASAP 2020 apparatus was employed to measure the specific surface area (SSA) and pore size distribution (PSD) of the powders. The measurements were carried out by nitrogen adsorption at liquid nitrogen temperature. The degassing temperature ranged from room temperature up to 200 °C for 20 min with a pressure range of 0–950 mmHg. Prior to measurement, the samples were degassed at 1.3 Pa at 200 °C. SSA values were calculated via the BET equation in the P/P^0^ range of 0.05–0.33. The Barrett Joyner Halenda (BJH) method was applied to calculate the PSD on isotherm branches.

The static water/photocatalyst/air contact angles were measured using the sessile droplet method on the surface of the sample pellets. The analysis was performed using a Drop Shape Analyzer DSA10Mk2 (Krüss, Germany). The pellet was obtained by pressing the photocatalyst at 7 kPa. A drop of water (5–6 μL) was deposited on the surface of the pellet using a syringe needle and pictures were immediately taken using a CCD camera. To measure the contact angles under UV light, a PLL UVA lamp (8 mW∙cm^−2^) was placed horizontally 10 cm above the pellets. After 10 min of excitation, the drop of water was deposited, and the lamp was pushed away just before instantly taking the pictures.

A HORIBA Jobin Yvon Lab RAM Aramis Raman spectrometer was used for Micro-Raman analysis. This spectrometer was equipped with a diode-pumped solid-state laser (operating at 785 nm). The signal collection was released on an air-cooled CCD multichannel, with an accuracy of ±1 cm^−1^ between 200 and 1000 nm.

Fluorescence spectra of ZnO and BZO were acquired by using a Jasco FP-6300 spectrofluorimeter (Jasco, Tokyo, Japan). Different amounts of samples (0.5, 1.0, and 2.0 g∙L^−1^) were dispersed in deionised water in an ultrasonic bath for 15 min and the suspensions were poured in 1 cm path quartz cell and irradiated with an excitation wavelength equal to 325 nm. The spectra were recorded in the wavelength range 360–600 nm with a scan rate of 100 nm/min, using equally wide (10 nm) excitation and emission slits. The deconvolution of the obtained spectra was performed by using the OriginPro^®^ 2021 suit of programs. Good fits with seven gaussian function peaks (R^2^ = 0.999) were obtained for the fluorescence spectra of both the ZnO and BZO (2.0 g∙L^−1^) suspensions, reported in the Supporting Information.

### 3.3. Solar Photocatalytic Experiments

The investigation of the 4–nitrophenol photodegradation (4–NP 98%, Sigma Aldrich) under natural solar irradiation was carried out utilizing a tubular Pyrex photoreactor (V = 100 mL). The experiments were carried out as described in our previous study [58]. Briefly, the reactor contained 80 mL of a 4-nitrophenol (4–NP) aqueous solution (20 mg∙L^−1^) with 80 mg of the tested photocatalyst. The used photocatalyst concentration (1 g L^−1^) allowed a safe comparison of the photoactivity of the photocatalysts, as above this value no further improvement of the degradation rate was observed. The tested samples were stirred in the dark for 1 h before exposure to natural sunlight, in order to reach adsorption/desorption equilibrium. The solar tests were performed, simultaneously, using three photoreactors containing bare ZnO, BZO, and TiO_2_ P25 (Evonik) during a sunny August day from 11 AM to 3 PM in the region of Gabes (South Tunisia, 33°53′024″ N 10°06′036″ E).

Since the three photoreactors were placed simultaneously and were irradiated under the same conditions, it was possible to develop a safe comparison of the photocatalytic performance of the ZnO, BZO, and P25 samples. Furthermore, reusability tests were also carried out for three consecutive days (verifying that the irradiation conditions did not change significantly), recovering the photocatalyst used in the previous test each time by centrifugation, washing it with distilled water and drying it at 70 °C overnight. At different time intervals, suspension samples were withdrawn and the solid separated using 0.2 µm Millipore filters (PTFE). The concentration of 4-NP was determined after measuring its absorption in the filtered solution using a Perkin Elmer 950 UV-vis spectrophotometer. The average intensity of sunlight during the photocatalytic runs was measured and was found to be 8.4 W∙m^−2^ between 315–400 nm (UV range) and 2900 µE∙m^−2^∙s^−1^ between 400–700 nm (visible range, where µE stands for micro-Einstein) [59].

## 4. Conclusions

Ba-doped ZnO photocatalysts and bare ZnO active under solar irradiation were synthesized by means of a simple precipitation method. XRD data revealed changes in lattice parameters and relative shifts of ZnO peaks due to the insertion of interstitial Ba^2+^ ions. UV-vis analyses showed a slight redshift of the spectra of the BZO sample compared to bare ZnO, thus indicating a slight improvement of the optical properties. The FTIR spectra showed an increased water adsorption capacity of the BZO sample. This was confirmed by contact angle measurements. Indeed, lower contact angles were measured, both in the dark and under UV light, from the shape of the water droplet deposited on the surface of the BZO photocatalyst. Surface hydrophilicity was correlated with the higher defectivity of the BZO sample, according to Raman and fluorescence spectroscopy results. Therefore, doping with Ba^2+^ ions confers a stronger hydrophilic character, which can be invoked, together with the improved optoelectronic properties often described in the literature, to account for the boosted solar photocatalytic activity. In fact, the observed kinetic constant values for the ZnO and BZO samples were 8∙10^−3^ and 11.3∙10^−3^ min^−1^, respectively, by assuming first order kinetics. The higher activity and the improved photocorrosion stability of the modified sample compared to the bare one suggest the use of Ba-doped ZnO as a promising candidate for the removal of hazardous substances present in diverse forms of polluted water, but also for its potential use in other types of reactions typical of heterogeneous photocatalysis, especially taking into account its stability against photocorrosion compared to bare ZnO.

## Figures and Tables

**Table 1:** Structural parameters, SSA, and E_g_ values of the synthesized photocatalysts.

Samples	a (Å)	c (Å)	V (Å^3^)	D (nm)	SSA_BET_ (m^2^∙g^−1^)	E_g_ (eV)
ZnO	3.2501 (1)	5.2068 (2)	47.633	32.6	1.9	3.25
BZO	3.2519 (1)	5.2098 (1)	47.714	22.7	27.3	3.22

## Data Availability

Data is contained within the article or supplementary material.

## Supplementary Materials

The following supporting information can be downloaded at: https://www.mdpi.com/article/10.3390/nano13202742/s1, Figure S1: 4-NP dark adsorption kinetics using bare ZnO (▶) and BZO (★) samples.

## References

[1] Lui J., Chen W.H., Tsang D.C.W., You S. (2020). A Critical Review on the Principles, Applications, and Challenges of Waste-to-Hydrogen Technologies. Renew. Sustain. Energy Rev..

[2] Pérez-Lucas G., El Aatik A., Aliste M., Navarro G., Fenoll J., Navarro S. (2023). Removal of Contaminants of Emerging Concern from a Wastewater Effluent by Solar-Driven Heterogeneous Photocatalysis: A Case Study of Pharmaceuticals. Water Air Soil Pollut..

[3] Berardinelli A., Hamrouni A., Dirè S., Ceccato R., Camera-Roda G., Ragni L., Palmisano L., Parrino F. (2021). Features and Application of Coupled Cold Plasma and Photocatalysis Processes for Decontamination of Water. Chemosphere.

[4] Ahmad T., Farooq U., Phul R. (2018). Fabrication and Photocatalytic Applications of Perovskite Materials with Special Emphasis on Alkali-Metal-Based Niobates and Tantalates. Ind. Eng. Chem. Res..

[5] Hisatomi T., Domen K. (2019). Reaction Systems for Solar Hydrogen Production via Water Splitting with Particulate Semiconductor Photocatalysts. Nat. Catal..

[6] Ulmer U., Dingle T., Duchesne P.N., Morris R.H., Tavasoli A., Wood T., Ozin G.A. (2019). Fundamentals and Applications of Photocatalytic CO_2_ Methanation. Nat. Commun..

[7] Parrino F., Camera Roda G., Loddo V., Palmisano L. (2016). Elemental Bromine Production by TiO_2_ Photocatalysis and/or Ozonation. Angew. Chem. Int. Ed..

[8] Abd-Elaal A., Parrino F., Ciriminna R., Loddo V., Palmisano L., Pagliaro M. (2015). Alcohol-Selective Oxidation in Water under Mild Conditions via a Novel Approach to Hybrid Composite Photocatalysts. ChemistryOpen.

[9] Khlifi H., Callone E., Parisi F., Sciascia L., Palmisano L., Dirè S., Parrino F. (2023). Amine-Stabilized Boronate Groups at the Surface of Modified TiO_2_ under Circumneutral pH Conditions: Application to Selective Photocatalytic Degradations. J. Photochem. Photobiol. A Chem..

[10] Kacem K., Casanova-Chafer J., Hamrouni A., Ameur S., Güell F., Nsib M.F., Llobet E. (2023). ZnO–TiO_2_/RGO Heterostructure for Enhanced Photodegradation of IC Dye under Natural Solar Light and Role of RGO in Surface Hydroxylation. Bull. Mater. Sci..

[11] Xin Z., He Q., Wang S., Han X., Fu Z., Xu X., Zhao X. (2022). Recent Progress in ZnO-Based Nanostructures for Photocatalytic Antimicrobial in Water Treatment: A Review. Appl. Sci..

[12] Motelica L., Vasile B.-S., Ficai A., Surdu A.-V., Ficai D., Oprea O.-C., Andronescu E., Jinga D.C., Holban A.M. (2022). Influence of the Alcohols on the ZnO Synthesis and Its Properties: The Photocatalytic and Antimicrobial Activities. Pharmaceutics.

[13] Motelica L., Oprea O.-C., Vasile B.-S., Ficai A., Ficai D., Andronescu E., Holban A.M. (2023). Antibacterial Activity of Solvothermal Obtained ZnO Nanoparticles with Different Morphology and Photocatalytic Activity against a Dye Mixture: Methylene Blue, Rhodamine B and Methyl Orange. Int. J. Mol. Sci..

[14] Popa M.L., Preda M.D., Neacșu I.A., Grumezescu A.M., Ginghină O. (2023). Traditional vs. Microfluidic Synthesis of ZnO Nanoparticles. Int. J. Mol. Sci..

[15] Náfrádi M., Alapi T., Veres B., Farkas L., Bencsik G., Janáky C. (2023). Comparison of TiO_2_ and ZnO for Heterogeneous Photocatalytic Activation of the Peroxydisulfate Ion in Trimethoprim Degradation. Materials.

[16] Lee K.M., Lai C.W., Ngai K.S., Juan J.C. (2016). Recent Developments of Zinc Oxide Based Photocatalyst in Water Treatment Technology: A Review. Water Res..

[17] Mochane M.J., Motloung M.T., Mokhena T.C., Mofokeng T.G. (2022). Morphology and Photocatalytic Activity of Zinc Oxide Reinforced Polymer Composites: A Mini Review. Catalysts.

[18] Qi K., Cheng B., Yu J., Ho W. (2017). Review on the Improvement of the Photocatalytic and Antibacterial Activities of ZnO. J. Alloys Compd..

[19] Lachheb H., Ajala F., Hamrouni A., Houas A., Parrino F., Palmisano L. (2017). Electron Transfer in ZnO-Fe_2_O_3_ Aqueous Slurry Systems and Its Effects on Visible Light Photocatalytic Activity. Catal. Sci. Technol..

[20] Ong C.B., Ng L.Y., Mohammad A.W. (2018). A Review of ZnO Nanoparticles as Solar Photocatalysts: Synthesis, Mechanisms and Applications. Renew. Sustain. Energy Rev..

[21] Hameed A.S.H., Karthikeyan C., Sasikumar S., Senthil Kumar V., Kumaresan S., Ravi G. (2013). Impact of Alkaline Metal Ions Mg^2+^, Ca^2+^, Sr^2+^ and Ba^2+^ on the Structural, Optical, Thermal and Antibacterial Properties of ZnO Nanoparticles Prepared by the Co-Precipitation Method. J. Mater. Chem. B.

[22] Peyghan A.A., Noei M. (2014). The Alkali and Alkaline Earth Metal Doped ZnO Nanotubes: DFT Studies. Phys. B Condens. Matter.

[23] Qiu X., Li L., Zheng J., Liu J., Sun X., Li G. (2008). Origin of the Enhanced Photocatalytic Activities of Semiconductors: A Case Study of ZnO Doped with Mg^2+^. J. Phys. Chem. C.

[24] Elhalil A., Elmoubarki R., Farnane M., Machrouhi A., Mahjoubi F.Z., Sadiq M., Qourzal S., Barka N. (2018). Synthesis, Characterization and Efficient Photocatalytic Activity of Novel Ca/ZnO-Al_2_O_3_ Nanomaterial. Mater. Today Commun..

[25] Modwi A., Khezami L., Taha K.K., Bessadok A.J., Mokraoui S. (2019). Photo-Degradation of a Mixture of Dyes Using Barium Doped ZnO Nanoparticles. J. Mater. Sci. Mater. Electron..

[26] Ahmad I., Ahmed E., Ahmad M., Akhtar M.S., Basharat M.A., Khan W.Q., Ghauri M.I., Ali A., Manzoor M.F. (2020). The Investigation of Hydrogen Evolution Using Ca Doped ZnO Catalysts under Visible Light Illumination. Mater. Sci. Semicond. Process..

[27] Oliveira A.G., de Lara Andrade J., Montanha M.C., Lima S.M., da Cunha Andrade L.H., Hechenleitner A.A., Pineda E.A., de Oliveira D.M. (2019). Decontamination and Disinfection of Wastewater by Photocatalysis under UV/Visible Light Using Nano-Catalysts Based on Ca-Doped ZnO. J. Environ. Manag..

[28] Hu H., Ji H.F., Sun Y. (2013). The effect of oxygen vacancies on water wettability of a ZnO surface. Phys. Chem. Chem. Phys..

[29] Bhamare V.S., Kulkarni R.M., Santhakumari B. (2019). 5% Barium Doped Zinc Oxide Semiconductor Nanoparticles for the Photocatalytic Degradation of Linezolid: Synthesis and Characterisation. SN Appl. Sci..

[30] Jayakrishnan A.R., Alex K.V., Tharakan A.T., Kamakshi K., Silva J.P.B., Prasad M.S., Sekhar K.C., Gomes M.J.M. (2020). Barium-Doped Zinc Oxide Thin Films as Highly Efficient and Reusable Photocatalysts. ChemistrySelect.

[31] Shirdel B., Behnajady M.A. (2020). Visible-Light-Induced Degradation of Rhodamine B by Ba Doped ZnO Nanoparticles.

[32] Shirdel B., Behnajady M.A. (2017). Sol-Gel Synthesis of Ba-Doped ZnO Nanoparticles with Enhanced Photocatalytic Activity in Degrading Rhodamine B under UV-A Irradiation. Optik.

[33] Sing K.S.W. (1982). Reporting Physisorption Data for Gas/Solid Systems. Pure Appl. Chem..

[34] Ali M., Ikram M., Ijaz M., Ul-Hamid A., Avais M., Anjum A.A. (2020). Green Synthesis and Evaluation of n-Type ZnO Nanoparticles Doped with Plant Extract for Use as Alternative Antibacterials. Appl. Nanosci..

[35] Modwi A., Taha K.K., Khezami L., Al-Ayed A.S., Al-Duaij O.K., Khairy M., Bououdina M. (2020). Structural and Electrical Characterization of Ba/ZnO Nanoparticles Fabricated by Co-Precipitation. J. Inorg. Organomet. Polym. Mater..

[36] N’konou K., Haris M., Lare Y., Baneto M., Napo K. (2016). Effect of Barium Doping on the Physical Properties of Zinc Oxide Nanoparticles Elaborated via Sonochemical Synthesis Method. Pramana.

[37] N’Konou K., Haris M., Lare Y., Baneto M., Napo K., Torchio P. (2016). Effect of Barium Doping on Structural and Optical Properties of Zinc Oxide Nanoparticles Synthesized by Microwave Hydrothermal Method. Phys. Status Solidi.

[38] Jaramillo A.F., Baez-Cruz R., Montoya L.F., Medinam C., Pérez-Tijerina E., Salazar F., Rojas D., Melendrez M.F. (2017). Estimation of the Surface Interaction Mechanism of ZnO Nanoparticles Modified with Organosilane Groups by Raman Spectroscopy. Ceram. Int..

[39] Calleja J.M., Cardona M. (1977). Resonant Raman Scattering in ZnO. Phys. Rev. B.

[40] Papadopoulou E.L., Zorba V., Pagkozidis A., Barberoglou M., Stratakis E., Fotakis C. (2009). Reversible Wettability of ZnO Nanostructured Thin Films Prepared by Pulsed Laser Deposition. Thin Solid Film.

[41] Fan X.M., Lian J.S., Zhao L., Liu Y.H. (2005). Single violet luminescence emitted from ZnO films obtained by oxidation of Zn film on quartz glass. Appl. Surf. Sci..

[42] Mishra S.H., Srivastava R.K., Prakashi S.G., Yadav R.S., Panday A.C. (2010). Photoluminescence and photoconductive characteristics of hydrothermally synthesized ZnO nanoparticles. Opto-Electron. Rev..

[43] Rodnyi P.A., Khodyuk I.V. (2011). Optical and luminescence properties of zinc oxide (Review). Opt. Spectrosc..

[44] Ghannam H., Chahboun A., Turmine M. (2019). Wettability of Zinc Oxide Nanorod Surfaces. RSC Adv..

[45] Huang K., Lü J., Zhang L., Tang Z., Yu J., Li P., Liu F. (2012). Effect of Magnesium Doping on the Light-Induced Hydrophilicity of ZnO Thin Films. J. Semicond..

[46] Mohamed K.M., Benitto J.J., Vijaya J.J., Bououdina M. (2023). Recent Advances in ZnO-Based Nanostructures for the Photocatalytic Degradation of Hazardous, Non-Biodegradable Medicines. Crystals.

[47] Abdul Hamid S.B., Teh S.J., Lai C.W. (2017). Photocatalytic Water Oxidation on ZnO: A Review. Catalysts.

[48] Parrino F., De Pasquale C., Palmisano L. (2019). Influence of Surface-Related Phenomena on Mechanism, Selectivity, and Conversion of TiO_2_-Induced Photocatalytic Reactions. ChemSusChem.

[49] Parrino F., Conte P., De Pasquale C., Laudicina V.A., Loddo V., Palmisano L. (2017). Influence of Adsorbed Water on the Activation Energy of Model Photocatalytic Reactions. J. Phys. Chem. C.

[50] Yadav V., Verma P., Sharma H., Tripathy S., Saini V.K. (2020). Photodegradation of 4-Nitrophenol over B-Doped TiO_2_ Nanostructure: Effect of Dopant Concentration, Kinetics, and Mechanism. Environ. Sci. Pollut. Res..

[51] Challagulla S., Payra S., Chakraborty C., Singh S.A., Roy S. (2019). Understanding the Role of Catalytic Active Sites for Heterogeneous Photocatalytic Oxidation of Methanol and Thermal Reduction of NO_x_. Mol. Catal..

[52] Weng B., Qi M.Y., Han C., Tang Z.R., Xu Y.J. (2019). Photocorrosion Inhibition of Semiconductor-Based Photocatalysts: Basic Principle, Current Development, and Future Perspective. ACS Catal..

[53] Liwu Z., Hanyun C., Ruilong Z., Yongfa Z. (2009). Photocorrosion Suppression of ZnO Nanoparticles via Hybridization with Graphite-like Carbon and Enhanced Photocatalytic Activity. J. Phys. Chem. C.

[54] Yu L., Chen W., Li D., Wang J., Shao Y., He M., Wang P., Zheng X. (2015). Inhibition of Photocorrosion and Photoactivity Enhancement for ZnO via Specific Hollow ZnO Core/ZnS Shell Structure. Appl. Catal. B Environ..

[55] Le A.T., Samsuddin N.S.B., Chiam S.L., Pung S.Y. (2021). Synergistic Effect of PH Solution and Photocorrosion of ZnO Particles on the Photocatalytic Degradation of Rhodamine B. Bull. Mater. Sci..

[56] Ishioka J., Kogure K., Ofuji K., Kawaguchi K., Jeem M., Kato T., Shibayama T., Watanabe S. (2017). In Situ Direct Observation of Photocorrosion in ZnO Crystals in Ionic Liquid Using a Laser-Equipped High-Voltage Electron Microscope. AIP Adv..

[57] Bokuniaeva A.O., Vorokh A.S. (2019). Estimation of Particle Size Using the Debye Equation and the Scherrer Formula for Polyphasic TiO_2_ Powder. J. Phys. Conf. Ser..

[58] Hamrouni A., Azzouzi H., Rayes A., Palmisano L., Ceccato R., Parrino F. (2020). Enhanced Solar Light Photocatalytic Activity of Ag Doped TiO_2_-Ag_3_PO_4_ Composites. Nanomaterials.

[59] Mõttus M., Sulev M., Baret F., Lopez-Lozano R., Reinart A., Meyers R.A. (2012). Photosynthetically Active Radiation: Measurement and Modeling. Encyclopedia of Sustainability Science and Technology.

