# Supplementary material for: Solar Photocatalytic Activity of Ba-Doped ZnO Nanoparticles: The Role of Surface Hydrophilicity
Source: Nanomaterials (Basel). 2023 Oct 10;13(20):2742. doi: 10.3390/nano13202742 (PMC10609520; doi:10.3390/nano13202742)
Supplement: Supplementary file 1 [file nanomaterials-13-02742-s001.zip › nanomaterials-2647425-supplementary.pdf]

Solar Photocatalytic Activity of Ba Doped ZnO Nanoparticles: The Role of Surface Hydrophilicity

Abdessalem Hamrouni [1,2], Marwa Moussa [1], Nidhal Fessi [1,3], Leonardo Palmisano [4], Riccardo Ceccato [5], Ali Rayes [1,2] and Francesco Parrino [5,*]

[1] Laboratory of Catalysis and Materials for the Environment and Processes LRCMEP (LR19ES08), Faculty of Sciences of Gabès/University of Gabès, University Campus Erriadh City, Gabès 6072, Tunisia hamrouni-28@hotmail.fr (A.H.); moussaamarwaa@gmail.com (M.M.); nidhal_fessi@hotmail.fr (N.F.); ali.rayes@fsb.rnu.tn (A.R.)

[2] Department of Chemical Engineering-Processes, National Engineering School of Gabès, Omar El Khateb Avenue, Zrig, Gabes 6029, Tunisia

[3] Laboratoire d'Automatique et de Génie des Procédés (LAGEPP), University of Lyon, UMR 5007 CNRS, University Claude Bernard Lyon 1, 69622 Villeurbanne, France

[4] Department of Engineering, University of Palermo, Viale delle Scienze Ed. 6, 90128 Palermo, Italy leonardo.palmisano@unipa.it

[5] Department of Industrial Engineering, University of Trento, Via Sommarive 9, 38123 Trento, Italy riccardo.ceccato@unitn.it

* Correspondence: francesco.parrino@unitn.it

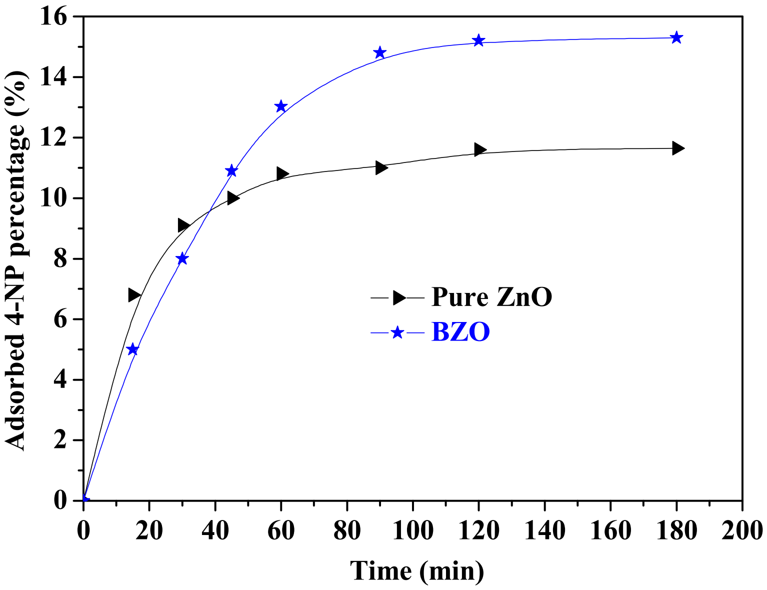

Figure S1: 4-NP dark adsorption kinetics using bare ZnO (▶) and BZO (★) samples.

Under dark conditions, the adsorbed quantity of 4-NP was not significant for both photocatalysts. About 11 % of the 4-NP was adsorbed in the presence of ZnO while BZO was capable to adsorb 15 %. These percentages are equivalent to adsorbed quantities equal to 28.31 $mg \cdot g^{-1}$ and to 39.74 $mg \cdot g^{-1}$, respectively. Notably, the larger SSA of BZO sample (about 14 times larger than ZnO) do not significantly affect the 4-NP adsorption in the dark.
